# Overexpression of the *NDR1/HIN1-Like* Gene *NHL6* Modifies Seed Germination in Response to Abscisic Acid and Abiotic Stresses in *Arabidopsis*

**DOI:** 10.1371/journal.pone.0148572

**Published:** 2016-02-05

**Authors:** Yan Bao, Wei-Meng Song, Jing Pan, Chun-Mei Jiang, Renu Srivastava, Bei Li, Lu-Ying Zhu, Hong-Yan Su, Xiao-Shu Gao, Hua Liu, Xiang Yu, Lei Yang, Xian-Hao Cheng, Hong-Xia Zhang

**Affiliations:** 1 College of Agriculture, Ludong University, 186 Hongqizhong Road, Yantai, 264025, China; 2 National Key Laboratory of Plant Molecular Genetics, Shanghai Institute of Plant Physiology and Ecology, Chinese Academy of Sciences, 300 Fenglin Road, Shanghai, 200032, China; 3 Plant Sciences Institute and the Department of Genetics, Development and Cell Biology, Iowa State University, Ames, IA, 50011, United States of America; 4 College of Life Sciences, Nanjing University, 22 Hankou Road, Nanjing, 210093, China; Instituto de Biología Molecular y Celular de Plantas, SPAIN

## Abstract

*NHL* (*NDR1/HIN1*-like) genes play crucial roles in pathogen induced plant responses to biotic stress. Here, we report the possible function of *NHL6* in plant response to abscisic acid (ABA) and abiotic stress. *NHL6* was highly expressed in non-germinated seeds, and its expression was strongly induced by ABA and multiple abiotic stress signals. Loss-of-function of *NHL6* decreased sensitivity to ABA in the early developmental stages including seed germination and post-germination seedling growth of the *nhl6* mutants. However, overexpression of *NHL6* increased sensitivity to ABA, salt and osmotic stress of the transgenic plants. Further studies indicated that the increased sensitivity in the *35S*::*NHL6* overexpressing plants could be a result of both ABA hypersensitivity and increased endogenous ABA accumulation under the stress conditions. It was also seen that the ABA-responsive element binding factors AREB1, AREB2 and ABF3 could regulate *NHL6* expression at transcriptional level. Our results indicate that *NHL6* plays an important role in the abiotic stresses-induced ABA signaling and biosynthesis, particularly during seed germination and early seedling development in *Arabidopsis*.

## Introduction

The plant hormone abscisic acid (ABA) plays major roles in the process of seed germination and plant adaptation to abiotic stresses [1–3]. Under stress conditions, expression of the ABA biosynthesis genes are induced, leading to enhanced synthesis of endogenous ABA [4]. Up to date, numerous *Arabidopsis* mutants that could germinate even in the presence of high concentrations of ABA have been identified. Among them, ABI1 (phosphatase ABA-INSENSITIVE 1) and ABI2 negatively regulate ABA signaling during seed dormancy and germination [5,6]. Whereas ABI3 (B3 type), ABI4 (AP2 type) and ABI5 (bZIP type, basic region leucine-zipper) are transcription factors that restrain growth when the germinating seeds confront adverse growth conditions [7–9]. PP2C (phosphatase 2C) cooperates with PYR (pyrabactin resistance)/PYL (PYR1-like), also called RCARs (regulatory components of ABA receptors), in ABA signal perception [10,11]. When plants encounter unfavorable environmental signals, endogenous ABA binds to PYR/PYL proteins and inhibits the phosphatase activity of ABI1 and ABI2, leading to the accumulation of phosphorylated protein kinase SnRK2s (Snf1-related protein kinase), followed by subsequent phosphorylation of ion channels or ABA-responsive transcriptional factors, such as AREB1, AREB2 and ABF3 [12,13].

In the *Arabidopsis* genome, 45 *NHL* (*NDR1/HIN1-like*) genes that are homologous to *NDR1* (non-race-specific disease resistance) or *HIN1* (harpin-induced) genes have been identified [14–17]. Their functions in pathogen perception have been extensively studied. Overexpression of *NHL2* in *Arabidopsis* resulted in elevated expression of *PR1* (pathogenesis-related gene 1) and light-dependent ‘speck disease-like’ symptoms in the leaves of transgenic plants [16]. Expression of *NHL3* and *NHL25* caused pathogen-dependent mRNA accumulation, while the overexpression of *NHL3* increased resistance to *Pseudomonas syringae* pv. *tomato* DC3000 in transgenic *Arabidopsis* [18]. *NHL10* was up-regulated during *Cucumber mosaic virus* infection [17]. Altered phloem export and sugar partitioning was seen in *Arabidopsis* as a result of higher expression of *NHL26* [19]. Interestingly, among these reported *NHL* genes, expression of *HIN1* and *NHL10* was also up-regulated in senescent leaves [17,20,21]. Since the process of leaf senescence mimicks the gradual dehydration of plants under drought stress condition [22], the possibility that *NHL* genes participate in ABA or abiotic stresses has been taken into account in our study.

We analyzed the published microarray data related to *NHL* genes in the *Arabidopsis* genome [23,24], and found that the expression level of both *NHL29* and *NHL6* increased more than three times in the senescent leaves or ABA treated seedlings (S1A Fig). *NHL29* is identical to HAB2 (Hypersensitive to ABA 2), one of the key components for ABA signal perception [25]. Therefore, we deduced that *NHL6* might also take part in ABA signaling in *Arabidopsis*. In this work, we provide direct evidence that *NHL6* is a key regulator for both ABA- and abiotic stress-mediated responses during seed germination and early seedling development in *Arabidopsis*.

## Materials and Methods

### Plant Growth and *nhl6* Mutant Isolation

All *Arabidopsis thaliana* materials are Columbia-0 (Col-0) ecotype. The T-DNA insertion mutants were bought from the *Arabidopsis* Biological Resource Center. Dr. Kazuo Shinozaki and Dr. Eiji Nambara from RIKEN Plant Science Center generously provided us the *abi1-1C* and *abi5-7* mutants. *abi3-8* was kindly provided by Prof. Chuanyou Li (Institute of Genetics and Developmental Biology, Chinese Academy of Sciences, Beijing, China). Plants were grown in the greenhouse as described previously [26,27,28]. For *nhl6* (SALK_148523) T-DNA insertion identification, the T-DNA border of *nhl6* was identified using the T-DNA left-border primer LBa1 and two *NHL6* gene-specific primers NHL6F and NHL6R (S1 Table). Homozygous *nhl6* mutant was identified by PCR to confirm the disruption of *NHL6* endogenous gene and RT-PCR to confirm the disruption of gene expression. *ACTIN2* was used as an internal control.

### NHL6 Promoter-β-Galactosidase (GUS) Construct and Histochemical Analyses

*NHL6* promoter-GUS construct was generated by amplifying the 1532 bp 5'-flanking DNA of *NHL6* coding region with the *NHL6* promoter specific primers PrNHL6-F and PrNHL6-R (S1 Table), and cloned into the pBlueScript SK- vector for sequence confirmation. The *Bam*HI-*Sal*I fragment from pBlueScript SK- subclone was inserted into the same sites in the pCAMBIA1381Z vector (pCAMBIA) to obtain the ProNHL6::GUS vector. The construct was transformed into wild-type *Arabidopsis* (Col-0 ecotype) plants as described previously [29].

For GUS expression assay, seedlings and different tissues from seedlings or plants at different developmental stages were collected and stained with 5-bromo-4-chloro-3-indolyl-D-glucuronide for 24 hours. They were then incubated in 75% ethanol to remove chlorophyll as described previously [30].

### Transgenic Vector and Plant Transformation

For *nhl6* mutant complementation, construct was generated by amplifying the 3.3 kb genomic DNA sequence including the 1532 bp DNA fragment upstream the predicted ATG start codon of *NHL6*. The full-length genomic sequence of *NHL6*, and the 527 bp 3’UTR was amplified by PCR from *Arabidopsis* genomic DNA with primers gNHL6-F and gNHL6-R, and cloned into the pCAMBIA1300 binary vector between the *Bam*HI and *Sal*I sites. The open reading frame of *NHL6* from the pBlueScript SK- subclone was inserted into a modified pCAMBIA2301 vector via the *Bam*HI and *Sal*I restriction sites driven by two copies of the *CaMV* 35S promoter, to be used as an overexpression vector. The resultant constructs were separately introduced into the *Agrobacterium GV3101* strain. Wild-type or *nhl6 Arabidopsis* plants were transformed as described previously [29]. Transgenic plants were screened on MS medium containing 50 μg/ml kanamycin or hygromycin. Primers used were listed in S1 Table.

### Germination Assay

We grew all the plants side by side to minimize the deviation. At least 100 seeds of each line were used for each experimental treatment, and three biological replicates were performed for statistical analyses. For each comparison, seeds were sown on the same plate containing MS medium with 2% sucrose and 0.8% agar, supplemented with or without different concentrations of ABA, NaCl or mannitol as indicated. Plates were stratified at 4°C for 3 d and moved to 22°C with 16h/light and 8h/dark cycles in a growth chamber. The percentage of seed germination was scored every 12 hours for 4 days. Germination rate was defined as the obvious emergence of radicles through the seed coats. The effect of ABA on germination greening is defined as that the cotyledons have obviously expanded and turned green. The data was recorded four days after they were moved to the growth chamber. Different batches of seeds were used in the germination assays.

### ABA Content Measurements

Four-day-old seedlings of wild-type, *nhl6* and *NHL6* overexpressing lines grown on MS medium supplemented with or without 200 mM NaCl or 400 mM mannitol were harvested at the same time. Plant materials were immediately put into liquid nitrogen and ground into fine powder, and then extracted in a buffer of 80% methanol, 0.5g/L citric acid and 0.1g/L butylated hydroxytoluence for 12 hours at 4°C. After centrifugation at 2000 rmp, the supernatant was vacuum dried and dissolved in Tris-saline buffer (25mM Tis, 100mM NaCl, 1mM MgCl_2_, pH 7.5). ABA concentration in the solution was determined using the Phytodetek ABA immunoassay kit (Agdia, Inc., Elkhart, IN) according to the manufacture’s protocol.

### RT-PCR and Quantitative Real-Time PCR Analyses

Gene expression levels were analyzed using quantitative real-time PCR (qRT-PCR) as described previously [28]. All the analysis was performed in the CFX Connect real-time PCR detection system using an AceQ qPCR SYBR Green Master Mix (Vazyme Biotech, China). Results were normalized to the reference gene *ACTIN2* using the ΔΔCt method. Each experiment was repeated three times. Primers used in this study are listed in S1 Table.

### Transient Transcription Dual-Luciferase Assay

To generate the ProNHL6::LUC reporter construct for the dual-luciferase assays, the 1532 bp promoter region of *NHL6* was digested from the pBlueScript SK- subclone and inserted into the *Bam*HI and *Sal*I sites of pGreenII0800-LUC. To generate the CaMV 35S promoter driven transcriptional factor effector constructs, *AREB1*, *AREB2*, *ABF3* or *ABI5* were digested from the pBlueScript SK- subclones and inserted into the *Bam*HI and *Sal*I sites of the pGreenII62-SK, respectively.

Transient dual-luciferase assays in *Arabidopsis* protoplasts were performedand checked using dual luciferase assay reagents (Promega, Shanghai, China) as described previously [28,31].

### NHL6 Subcellular Localization and Co-Localizaion Analyses

Foe subcellular localization of NHL6, N-terminal fusion construct 1300S-YFP-NHL6 was introduced into *Agrobacterium tumefaciens* strain *GV3101* and infiltrated into the leaves of *N*. *benthamiana*. After 48 hours, the epidermis of infiltrated leaves was examined for YFP signals under a Zeiss 510META confocal laser scanning microscope. The filter settings are as follows: YFP: Ex 514 nm / Em BP 535 to 600 nm; chlorophyll: Ex 488 nm / Em LP 650 nm.

The GFP-NHL6 and GFP-AtTLP11 fusion protein were transiently expressed in *Arabidopsis* protoplasts [32,33]. For co-localization of NHL6 and AtTLP11, GFP and mCherry were respectively fused to the N-terminal and C-terminal to result in the GFP-NHL6 and AtTLP11-mCherry vectors. Equal amounts of GFP-NHL6 and AtTLP11-mCherry plasmids were used for co-transformation. The transformed *Arabidopsis* protoplasts were analyzed at wavelength 488 nm for GFP-NHL6 or GFP-AtTLP11 expression and at wavelength 559 nm for AtTLP11-mCherry expression using a confocal microscope (Olympus FluoView1000, confocal microscope). pA7-GFP was used as a positive control [34].

### Statistics

All data in this work were obtained from at least three independent experiments with three replicates each. Data were analyzed using one-way analysis of variance (ANOVA) followed by Duncan's multiple range test (P <0.01).

## Results

### *NHL6* Is Induced by ABA and Different Stress Treatments

Like the *NDR1*/*HIN1* genes, the expression of most *NHL* genes is induced by biotic stress such as pathogen infection [16–18]. To explore the possible roles of *NHL* family genes in plant response to abiotic stresses, we searched the published microarray data [23,24] and found 45 *NHLs* in the *Arabidopsis* genome. Different from the other family members, *NHL6* showed very high expression in senescent leaves (S1A, S1B and S1D Fig), and was strongly induced by ABA (S1A and S1C Fig). Since ABA plays a prominent role in abiotic stress adaptation and senescence, we assumed that *NHL6* might function in plant response to ABA and abiotic stress.

**Fig 1:**
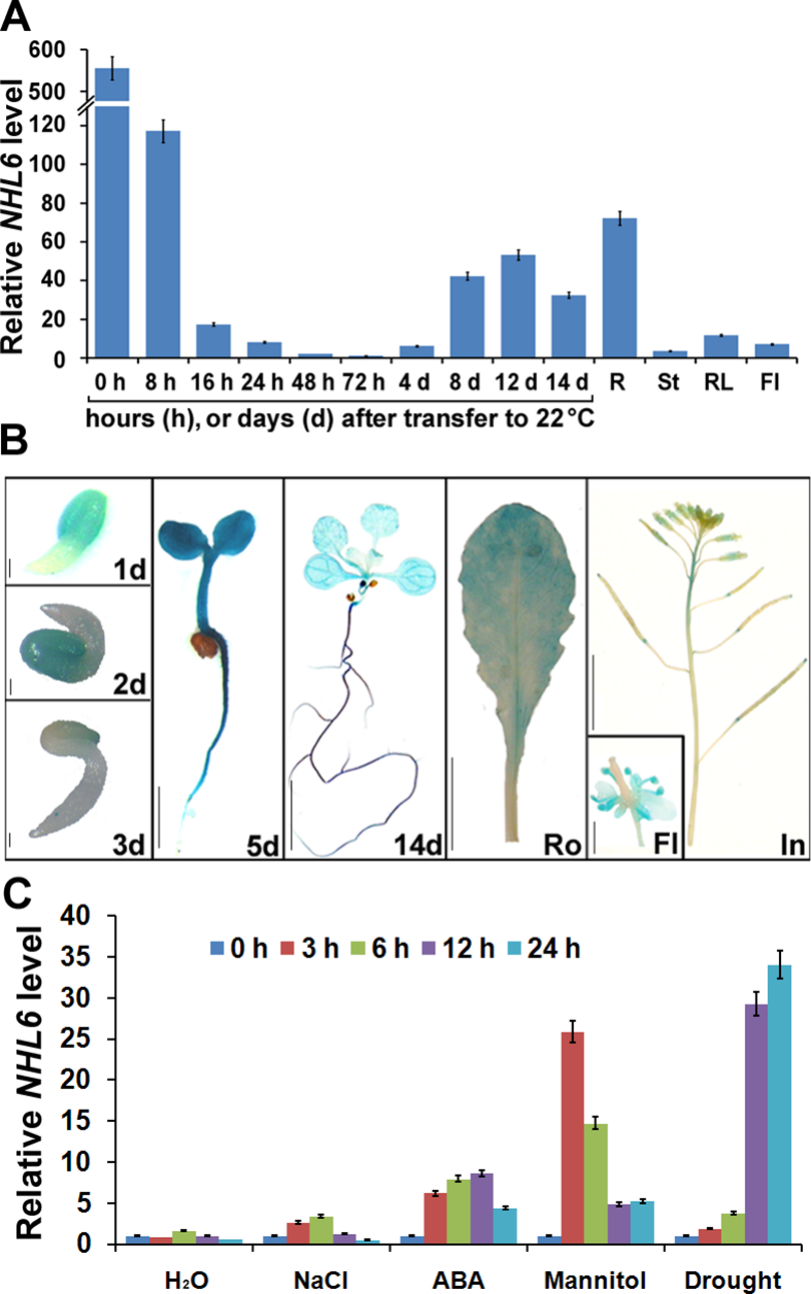
Expression pattern of *NHL6* in wild-type (Col-0) *Arabidopsis*. (A) qRT-PCR analyses of *NHL6* transcripts. cDNA derived from germinating seeds and the indicated organs of six-week-old wild-type plants was used as templates. Seeds were floated on MS medium and vernalized at 4°C for 3 days before being sowed. *ACTIN2* was used as a reference gene for normalization of expression and the transcript level in 72-hour-old seedlings was set as 1.0. Error bars represent SD (*n* = 3). R, root; St, stem; RL, rosette leaf; Fl, flower. (B) ProNHL6-β-glucuronidase (GUS) expression in transgenic *Arabidopsis* plants. Histochemical GUS staining was carried out at different germinating stages and in various tissues of *NHL6* promoter-GUS transgenic plants. 1d, an one-day-old seedling, scale bar = 0.1 mm; 2d, a two-day-old seedling, scale bar = 0.1 mm; 3d, a three-day-old seedling, scale bar = 0.1 mm; 5d, a five-day-old seedling, scale bar = 2 mm; 14d, a fourteen-day-old seedling, scale bar = 2 mm; Ro, a rosette leaf, scale bar = 5 mm; In, an inflorescence, scale bar = 5 mm; Fl, a flower, scale bar = 1 mm. (C) qRT-PCR analyses of *NHL6* expression were conducted after treatments with distilled water (H_2_O), 200 mM NaCl, 100 μM ABA, 400mM mannitol or drought for indicated time intervals. *ACTIN2* was used as a reference gene for normalization of expression and the transcript level in untreated seedlings at 0 hour was set as 1.0. Error bars represent SD (*n* = 3). Two-week-old seedlings grown on MS media were used for the treatments.

As a first step to understand the possible biological function of *NHL6*, we performed qRT-PCR (quantitative real-time reverse transcription-PCR) and examined its expression pattern in wild-type *Arabidopsis*. *NHL6* is ubiquitously expressed in seedlings and various tissues, including roots, stems, rosette leaves and flowers (Fig 1A). However, the most abundant *NHL6* mRNA was present in seeds, roots and senescent leaves (Fig 1A and S1D Fig). To assess the promoter activity, we also generated *NHL6* promoter-β-glucuronidase (GUS) reporter vector and introduced it into wild-type *Arabidopsis* plants. Consistent with the qRT-PCR results, GUS was also expressed in seedlings and various tissues (Fig 1B).

The microarray data showed that *NHL6* expression can be induced by ABA (S1A and S1C Fig). Therefore, we examined the expression level of *NHL6* after ABA and different stress treatments. qRT-PCR analyses demonstrated that expression of *NHL6* was strongly induced by ABA, mannitol and drought, and slightly induced by NaCl (Fig 1C). All these results suggest that *NHL6* is an ABA and abiotic stress inducible gene.

### *NHL6* Knockout Mutant Is Insensitive to ABA during Seed Germination

**Fig 2:**
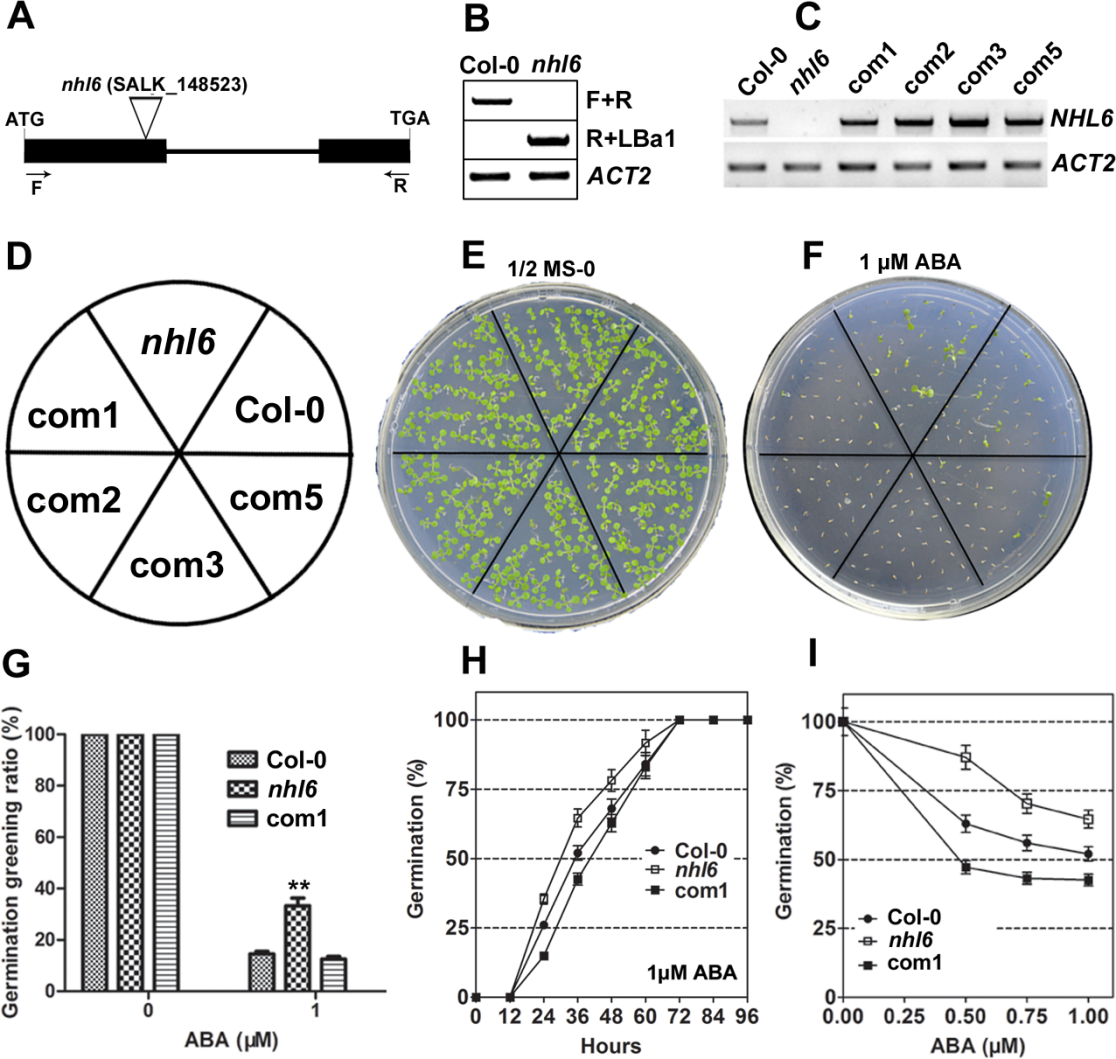
Molecular identification and ABA insensitivity of *nhl6* mutant. (A) Schematic diagram of T-DNA insertion in *nhl6* mutant. Exons and intron are depicted to scale by boxes and line, respectively. The coding region of *NHL6* is shown as black boxes. The position of T-DNA is indicated by triangle. (B) PCR analysis to confirm the disruption of *NHL6* gene by T-DNA insertion. The positions of primers F and R are indicated in (A). *ACTIN2* was used as a native control. (C) RT-PCR analyses of *NHL6* gene expression in wild-type Col-0, homozygous *nhl6* knockout mutant, and the complemented *ProNHL6*::*NHL6*/*nhl6* transgenic lines 1, 2, 3 and 5 (com1, 2, 3, 5). Total RNA was isolated from the rosette leaves of four-week-old plants. Expression of *ACTIN2* (*ACT2*) was analyzed as an internal control. (D-F) Seeds of Col-0, *nhl6* and the complemented *ProNHL6*::*NHL6*/*nhl6* transgenic lines 1, 2, 3 and 5 were sowed on MS medium supplemented with or without 1μM ABA. Photographs were taken after one week. (G) Statistical analyses of seed germination greening ratio in Col-0, *nhl6* and *ProNHL6*::*NHL6*/*nhl6* transgenic line 1 (com1). After the vernalization, seeds on MS medium plates were transferred to a growth chamber and cultured for 7 days. Values are means ±SE, n = 3 independent experiments. (H) Germination time course (hours after incubation at 22°C) of Col-0, *nhl6* and *ProNHL6*::*NHL6*/*nhl6* transgenic line 1 on MS medium supplemented with 1 μM ABA. (I) Germination rates of Col-0, *nhl6* and *ProNHL6*::*NHL6*/*nhl6* transgenic line 1 after 2 days at 22°C in the presence of different concentrations of ABA. Col-0, closed circles; *nhl6*, open squares; com1, closed squares. Results are presented as average values and standard errors from three experiments. At each time, at least 100 seeds were counted.

To further determine the possible function of *NHL6* in *Arabidopsis*, we ordered a mutant line (*nhl6*) that harbors a T-DNA insertion in the first exon of *NHL6* from ABRC (*Arabidopsis* Biological Resource Center) (Fig 2A). Homozygous *nhl6* mutant plants were first identified by PCR with genomic DNA as template (Fig 2B). As confirmed by RT-PCR analyses using *NHL6*-specific primers, T-DNA insertion abolished the expression of *NHL6* in *nhl6* (Fig 2C).

When grown on normal MS medium, *nhl6* plants did not show any visible phenotypic changes. The germination and subsequent growth of *nhl6* seedlings were similar to that of the wild-type plants (Fig 2E). However, *nhl6* was insensitive to ABA treatment compared to wild-type. Germination greening ratio (Fig 2D–2G) and germination rate (Fig 2H) in the wild-type were significantly inhibited by 1 μM ABA, but the inhibition in *nhl6* was less severe. More detailed analyses of germination rates under different concentrations of ABA were performed. Germination of wild-type seeds was significantly inhibited at 0.5μM ABA, more than 85% of *nhl6*, but less than 65% of wild-type seeds germinated (Fig 2I). To determine whether the observed phenotypic change was indeed caused by the disrupted expression of *NHL6*, we generated four independent complementary lines (Fig 2C). A 3.3 kb genomic DNA fragment containing the complete coding region (with intron) and putative promoter of *NHL6* was introduced into homozygous *nhl6* plants. Such a genomic fragment restored the expression of *NHL6* and rescued the ABA-insensitive phenotype in all *nhl6* complementary lines (Fig 2F). Therefore, complementary line 1 (com1) was chosen for detailed germination greening ratio (Fig 2G) and germination rate assays (Fig 2H and 2I). All these results from the complementation analyses confirmed that the observed mutant phenotypes were due to the disruption of *NHL6* gene.

### Transgenic Plants Overexpressing *NHL6* Are Hypersensitive to ABA, NaCl and Osmotic Stress

**Fig 3:**
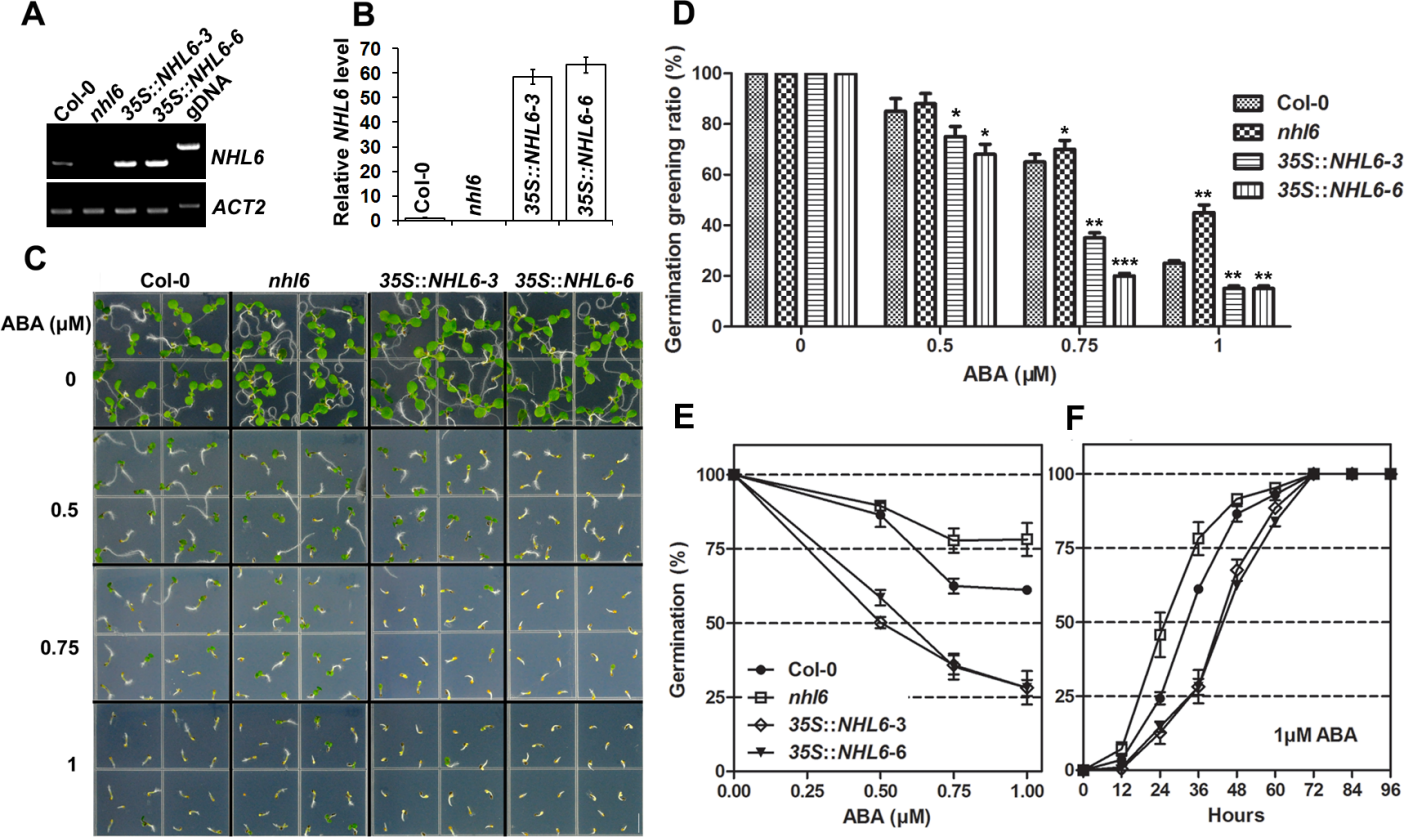
Hypersensitivity of transgenic plants overexpressing *NHL6*. (A, B) RT-PCR and qRT-PCR analyses of *NHL6* expression level in Col-0, *nhl6* and *35S*::*NHL6* overexpressor lines 3 and 6. *ACTIN2* was used as a reference gene for normalisation of expression and the transcript level in Col-0 was set as 1.0. Error bars represent SD (*n* = 3). (C, D) ABA-mediated inhibition of seed germination greening. Seeds of Col-0, *nhl6* and *35S*::*NHL6* overexpressor lines 3 and 6 were vernalized at 4°C for three days on MS medium, and then transferred to a growth chamber. Photographs were taken after one week. Scale bar = 5 mm. (E) Germination rates on MS medium supplemented with different concentrations of ABA for 2 days. (F) germination time course on MS medium supplemented with 1 μM ABA. Col-0, closed circles; *nhl6*, open squares, *35S*::*NHL6-3*, open rhombuses; *35S*::*NHL6-6*, closed triangles. Results are presented as average values and standard errors from three experiments. At each time, at least 100 seeds were counted.

To further understand the function of *NHL6*, we produced transgenic *Arabidopsis* plants overexpressing *NHL6*. Expression of *NHL6* in transgenic plants was driven by the 35S cauliflower mosaic virus (CaMV) promoter [35]. At least twenty independent transgenic lines (T_1_ generation) were obtained. Ten lines were grown to produce seeds and all transgenic lines showed similar growth phenotype on MS medium when compared with the wild-type plants under normal condition. Therefore, two representative homozygous lines (3 and 6) were chosen for further experiments. RT-PCR and qRT-PCR analyses confirmed the overexpression of *NHL6* in both transgenic lines (Fig 3A and 3B). *NHL6* overexpression did not cause any significant phenotypic changes in the transgenic plants when grown on normal MS medium (Fig 3C) as seen previously in the *nhl6* mutant. However, seeds of both transgenic lines (3 and 6) were hypersensitive to ABA as compared to the wild-type (Fig 3C, 3D and 3E). The time course of germination was also determined, and the results further supported the conclusion that mutation of *nhl6* renders the seedlings insensitive while overexpression of *NHL6* renders the seedlings hypersensitive to ABA (Fig 3F). Therefore, *NHL6* appears to act as a positive regulator in ABA-mediated seed germination inhibition.

**Fig 4:**
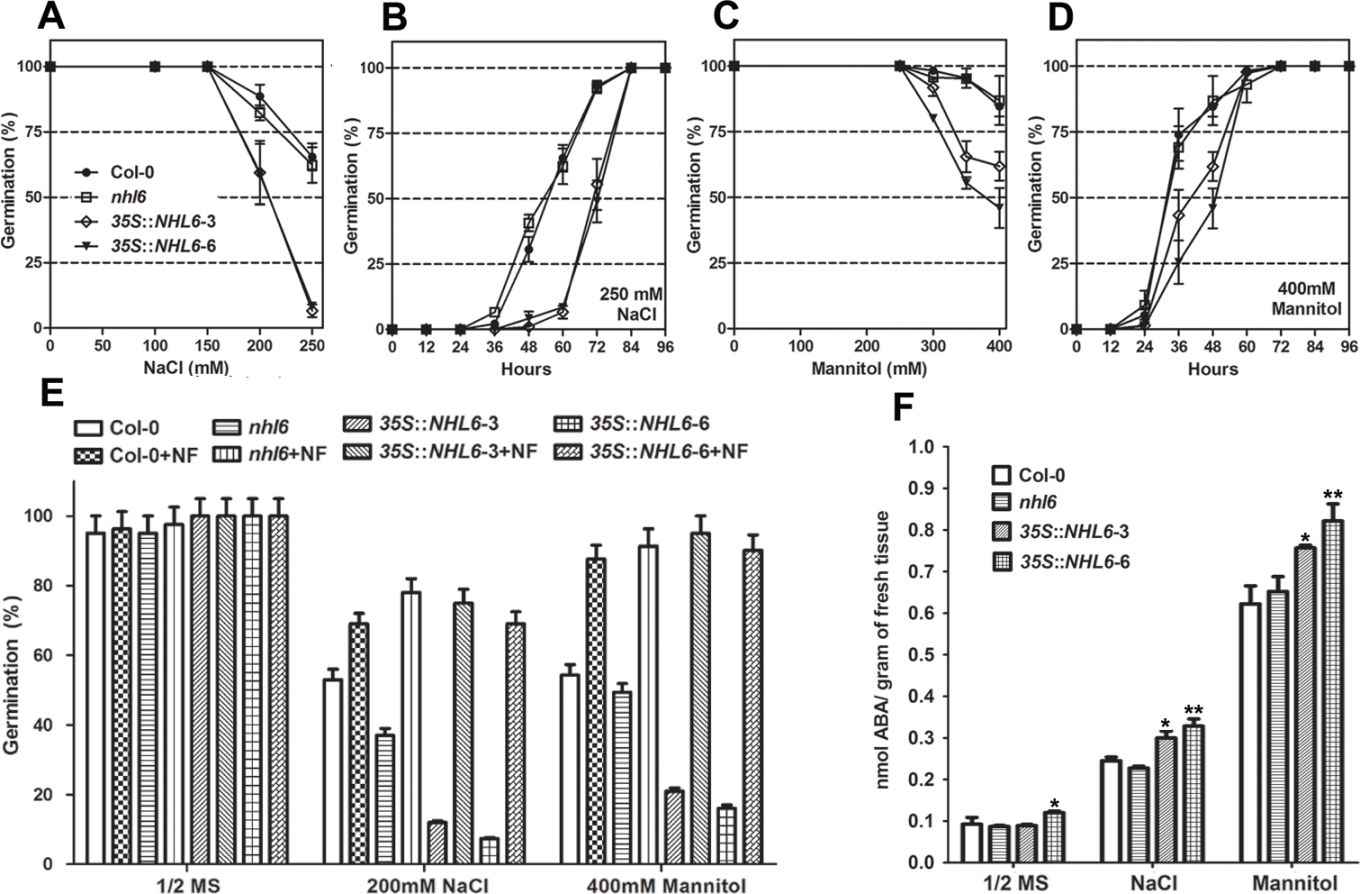
Germination and abiotic stress-mediated ABA accumulation in Col-0, *nhl6* and *35S*::*NHL6* overexpressor lines. (A, C) Germination rates on MS medium supplemented with different concentrations of NaCl or mannitol for 2 days. (B, D) Germination time course on MS medium supplemented with 250mM NaCl or 400mM mannitol. Col-0, closed circles; *nhl6*, open squares, *35S*::*NHL6-3*, open rhombuses; *35S*::*NHL6-6*, closed triangles. Results are presented as average values and standard errors from three experiments. (E) Germination rates on MS medium supplemented with 100 μM NF (norflurazon, an inhibitor of ABA biosynthesis) and 200 mM NaCl or 400 mM mannitol. Seed germination rates were scored after 2 days at 22°C. Results are presented as average values and standard errors from three experiments. At each time, at least 100 seeds were counted. (F) ABA content assays in four-day-old seedlings of Col-0, *nhl6* and *35S*::*NHL6* overexpressor lines grown on MS medium or MS medium supplemented with 200mM NaCl or 400 mM mannitol. Results shown are means of two independent experiments. Asterisks indicate significant differences from the corresponding control values at *0.01 < P < 0.05 and **P < 0.01 using the Student’s *t*-test.

*NHL6* expression is not only induced by ABA, but also slightly induced by salt and highly induced by mannitol and drought treatments (Fig 1C). Therefore, we examined the seed germination rates of wild-type, *nhl6* and *35S*::*NHL6* overexpression lines on MS medium supplemented with different concentrations of NaCl and mannitol. Although the germination rate of *nhl6* seeds was similar to that of wild-type seeds (Fig 4A–4D), *35S*::*NHL6* seeds were hypersensitive to high salt (250 mM NaCl) (Fig 4A and 4B) and osmotic stress (400mM mannitol) (Fig 4C and 4D).

### Overexpression of *NHL6* Stimulates Endogenous ABA Production

**Fig 5:**
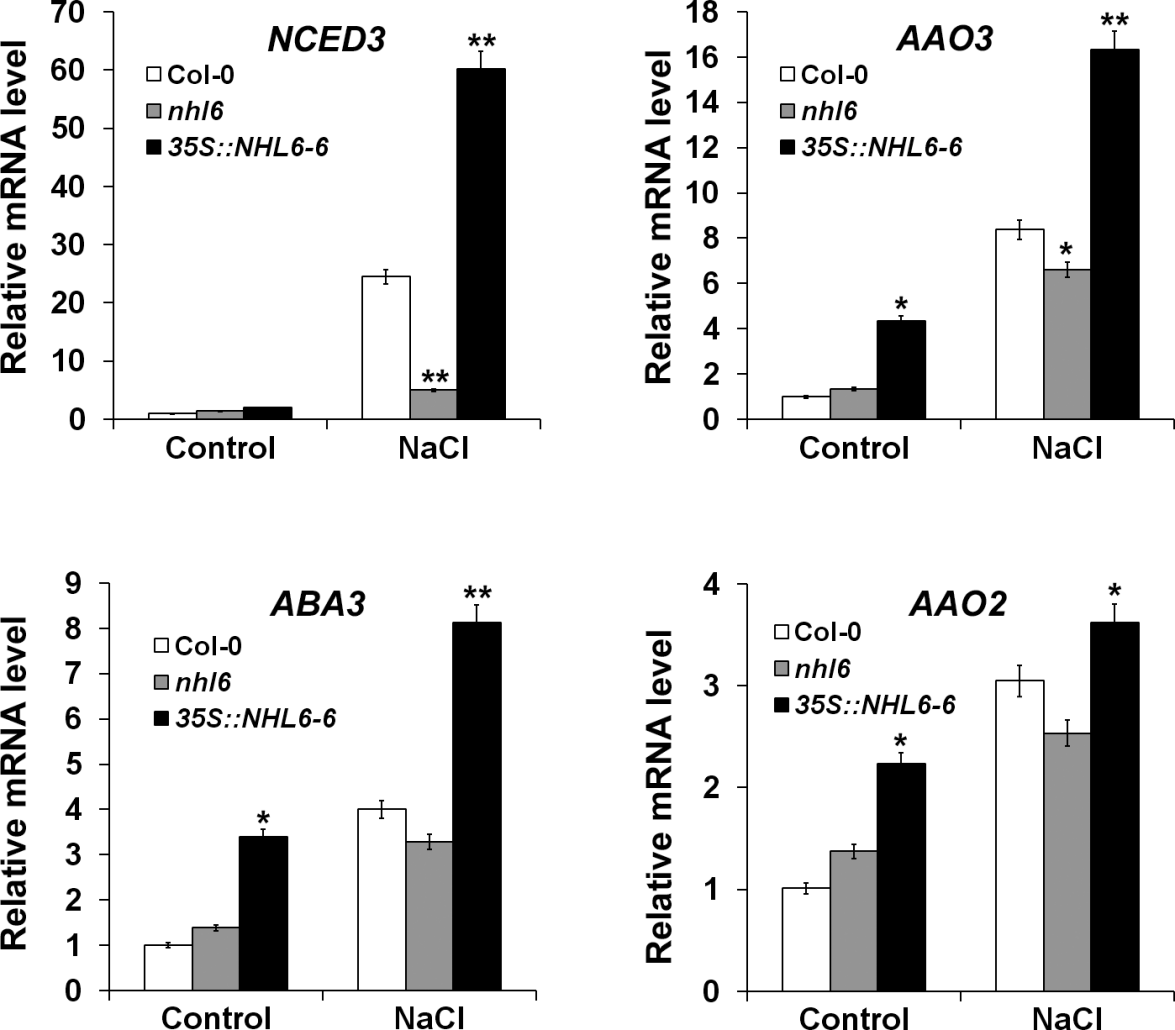
qRT-PCR analyses of ABA biosynthesis genes in Col-0, *nhl6* and *35S*::*NHL6* overexpressor line 6. Seven-day-old seedlings grown on MS were treated with water or 200 mM NaCl for 6 hours. The expression levels of ABA biosynthesis genes *NCED3*, *AAO3*, *ABA3* and *AAO2* were examined. *ACTIN2* was used as a reference gene for normalisation of expression and the transcript level in untreated Col-0 was set as 1.0. Error bars represent SD (*n* = 3). Asterisks indicate significant differences from the corresponding control values at *0.01 < P < 0.05 and **P < 0.01 using the Student’s *t*-test. (*n* = 3).

It has been well documented that high salt or hyperosmotic stress can induce ABA production in plants [1,36]. Therefore, the hypersensitive response of *35S*::*NHL6* seeds to these abiotic stresses could be caused by improved endogenous ABA accumulation. To understand whether this is an ABA-dependent process, norflurazon (NF) (Supelco, Bellefonte, PA, USA), an inhibitor for ABA biosynthesis, was added into the germination medium. Under normal condition, 100 mM NF did not affect the germination of wild-type (Col-0), *nhl6* or *35S*::*NHL6* seeds in a six-day germination assay (Fig 4E). However, on medium supplemented with 200 mM NaCl or 400 mM mannitol, seed germination of *35S*::*NHL6* was severely inhibited. Only 50% of wild-type seeds, and less than 15% and 22% of the *35S*::*NHL6* seeds germinated on the salt and mannitol medium (Fig 4E). By contrast, in the presence of 100 mM NF, the germination rates of wild-type, *nhl6* and the *35S*::*NHL6* seeds under the NaCl and mannitol exposure were restored to more than 73% and 90%, respectively (Fig 4E). These results suggested that salt and osmotic stress exerted their inhibitory effect on *35S*::*NHL6* seed germination through ABA biosynthesis. Therefore, we further measured ABA content and ABA biosynthesis gene expression in the four-day-old seedlings of wild-type, *nhl6*, and *35S*::*NHL6* grown on MS medium or MS medium supplemented with 200 mM NaCl or 400 mM mannitol. Indeed, compared to the wild-type and *nhl6*, both *35S*::*NHL6-3* and *35S*::*NHL6-6* accumulated more ABA upon treatment with 200 mM NaCl or 400 mM mannitol (Fig 4F). And, the expression levels of multiple ABA biosynthesis genes were significantly higher in the *35S*::*NHL6* overexpression plants, especially after they were treated with salt stress (Fig 5). These results indicated that the hypersensitive response to salt and osmotic stresses of *NHL6* overexpression lines is ABA-dependent. Therefore, *NHL6* plays a role not only in the regulation of ABA sensitivity, but also in the biosynthesis of ABA under abiotic stress conditions.

### *NHL6* Is Transcriptionally Regulated by AREB1, AREB2 and ABF3

**Fig 6:**
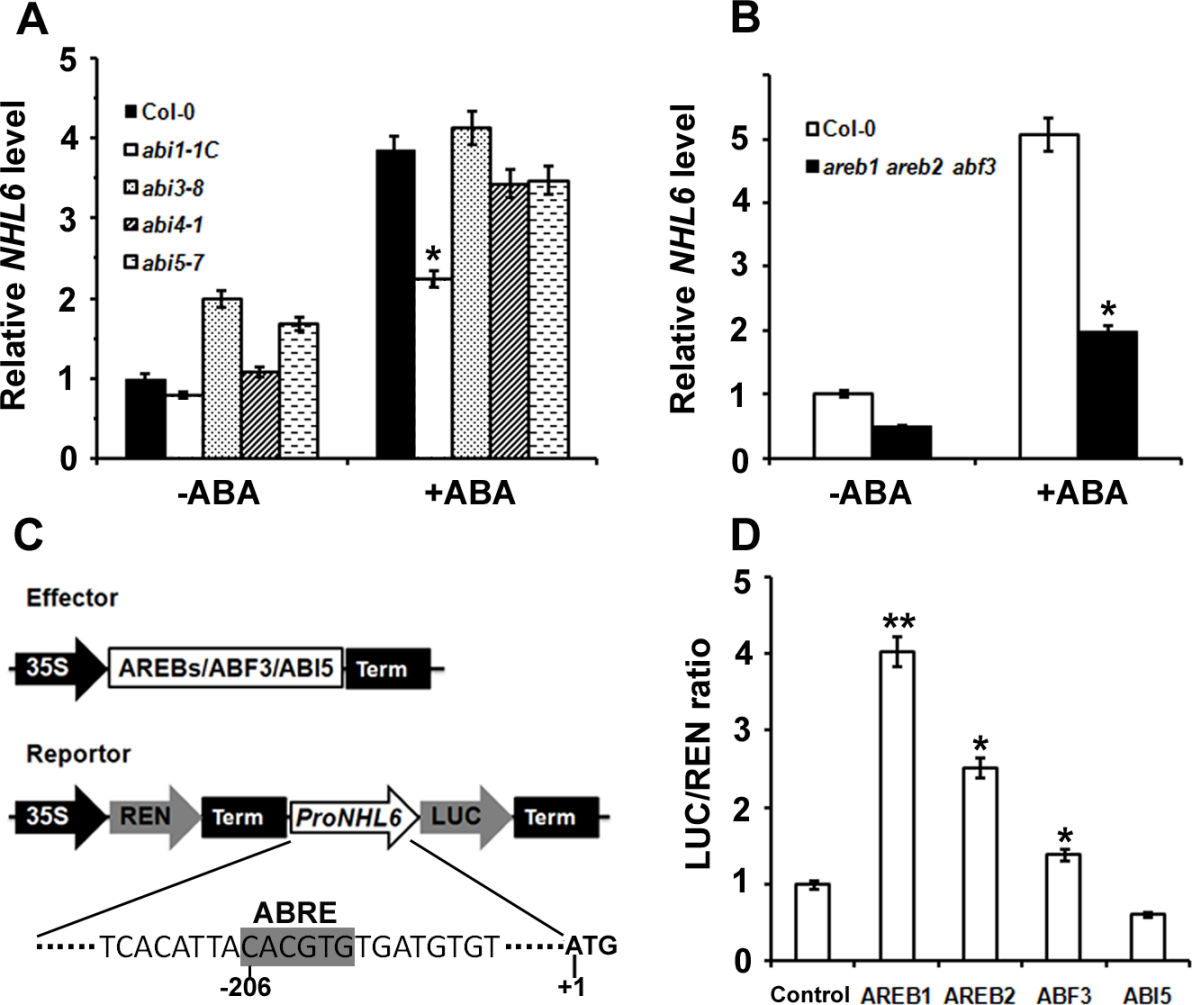
AREBs and ABF3 activated the expression of *NHL6*. (A) *NHL6* mRNA levels in various ABA signal deficient mutants. Two-week-old seedlings grown on MS medium were treated with 100 μM ABA for 6 hours. *NHL6* expression was examined by qRT-PCR analyses after the treatments. *ACTIN2* was used as a reference gene for normalisation of expression and the transcript level in the untreated Col-0 seedlings was set as 1.0. Error bars represent SD (*n* = 3). (B) ABA induced *NHL6* expression was significantly blocked in the *areb1areb2abf3* triple mutant (base on the published data by Yoshida *et al*. 2010). (C) Schematic diagram of the vector structure of effectors and reporter used in transient assays. (D) Transient assays of ProNHL6::LUC expression. 35S::REN-NHL6::LUC reporter construct was transiently expressed in Arabidopsis protoplasts together with the control vector, 35S::AREB1, 35S::AREB2, 35S::ABF3 or 35S::ABI5 effectors, respectively. The expression level of REN was used as an internal control. LUC/REN ratio represents the relative activity of the *NHL6* promoter. Data are values of three independent experiments. Asterisks indicate significant differences from the corresponding control values at *0.01 < P < 0.05 and **P < 0.01 using the Student’s *t*-test. (*n* = 3).

To understand whether *NHL6* is involved in ABA signal transduction, we investigated the expression level of *NHL6* in ABA signal deficient mutants. We found that compared to the wild-type (Col-0), *abi3-8*, *abi4-1* and *abi5-7* mutants, ABA induced *NHL6* expression was evidently cut down in the *abi1-1C* mutant (Fig 6A), implying that ABA signal transduction mediated by *ABI1* is required for ABA induced expression of *NHL6*.

Transcriptional factors AREB1, AREB2 and ABF3 have been reported to collaborate with each other to regulate ABA signal transduction through binding to the ABRE (ABA-responsive element) motifs in the promoter region of their target genes [13]. We found that ABA induced *NHL6* expression was enormously reduced in the *areb1areb2abf3* triple mutant (Fig 6B). By searching through PlantCare, a database of plant *cis*-acting regulatory elements and a portal to tools for in silico analysis of promoter sequences [37], one ABRE motif was identified in the promoter region of *NHL6*. Therefore, we postulated that transcription of *NHL6* may be regulated by these transcriptional factors. To verify this postulation, we performed transient transcriptional activity assays in *Arabidopsis* protoplasts using *ProNHL6*::LUC (firefly luciferase-coding gene driven by the *NHL6* promoter) as a reporter, and *AREB1*, *AREB2*, *ABF3* or *ABI5* (negative control) driven by the 35S promoter as effectors (Fig 6C). We found that AREB1, AREB2 and ABF3, especially AREB1, all induced the expression of LUC, but ABI5 did not (Fig 6D). These results suggest that AREB1, AREB2 and ABF3 could regulate the expression of *NHL6*.

### NHL6 Moves from Plasma Membrane to Cytosol in Response to ABA

**Fig 7:**
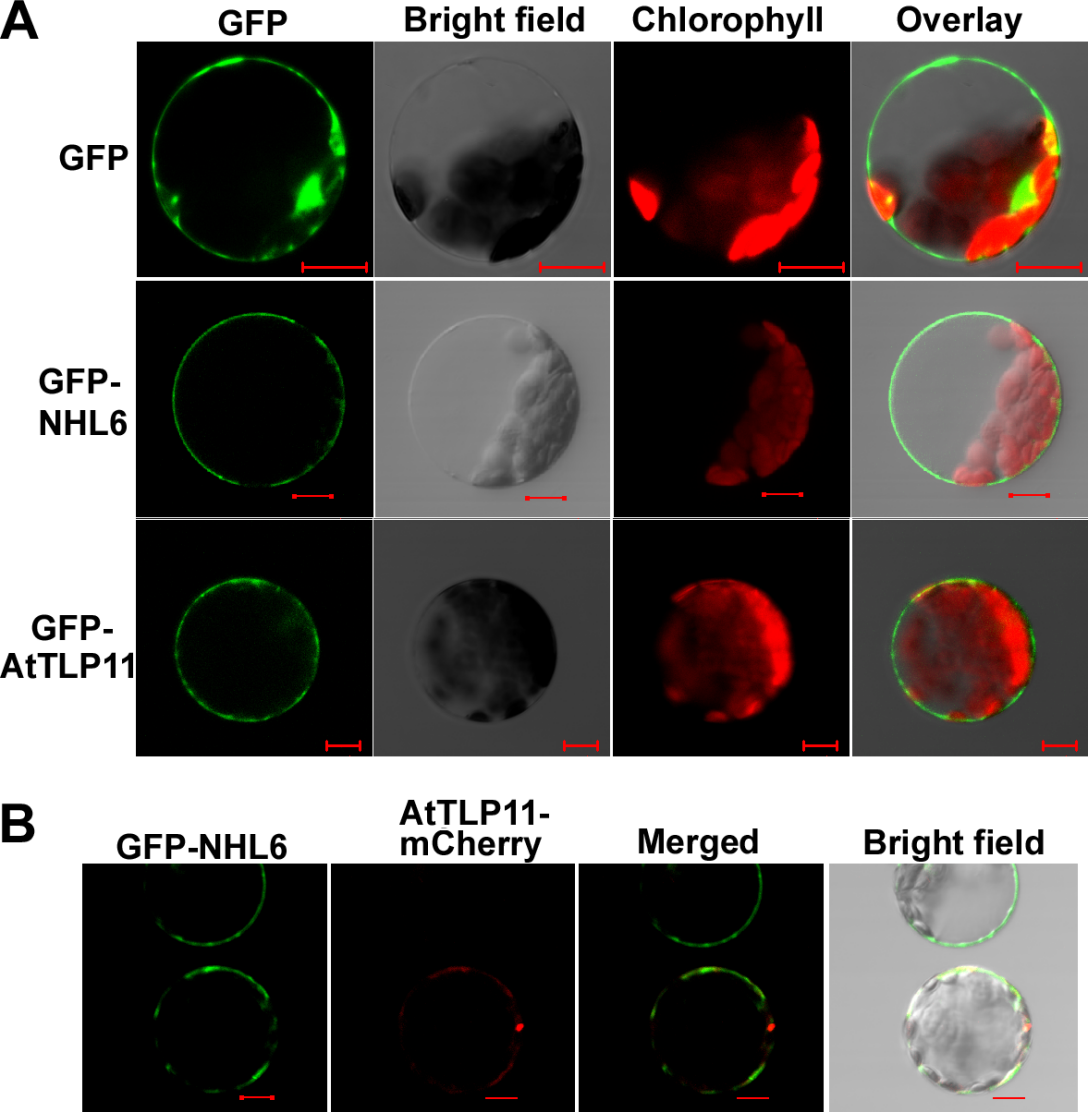
Subcellular localization and co-localization of NHL6 and AtTLP11. (A) Confocal microscopic analyses of GFP signals from Arabidopsis mesophyll protoplasts transiently expressing GFP, GFP-NHL6 or GFP-AtTLP11 fusion protein as indicated. Images of the GFP signal (green), bright field, chlorophyll fluorescence (red), and overlay (green plus red) from the same cell were shown. Scale bar = 10 μm. (B) Confocal microscopic analyses of GFP or mCherry signals from Arabidopsis mesophyll protoplasts transiently co-expressing GFP-NHL6 and AtTLP11-mCherry fusion proteins. Images of GFP signal (green), mCherry fluorescence (red), merged (green plus red) and overlay from the same cell were shown. Scale bar = 10 μm.

**Fig 8:**
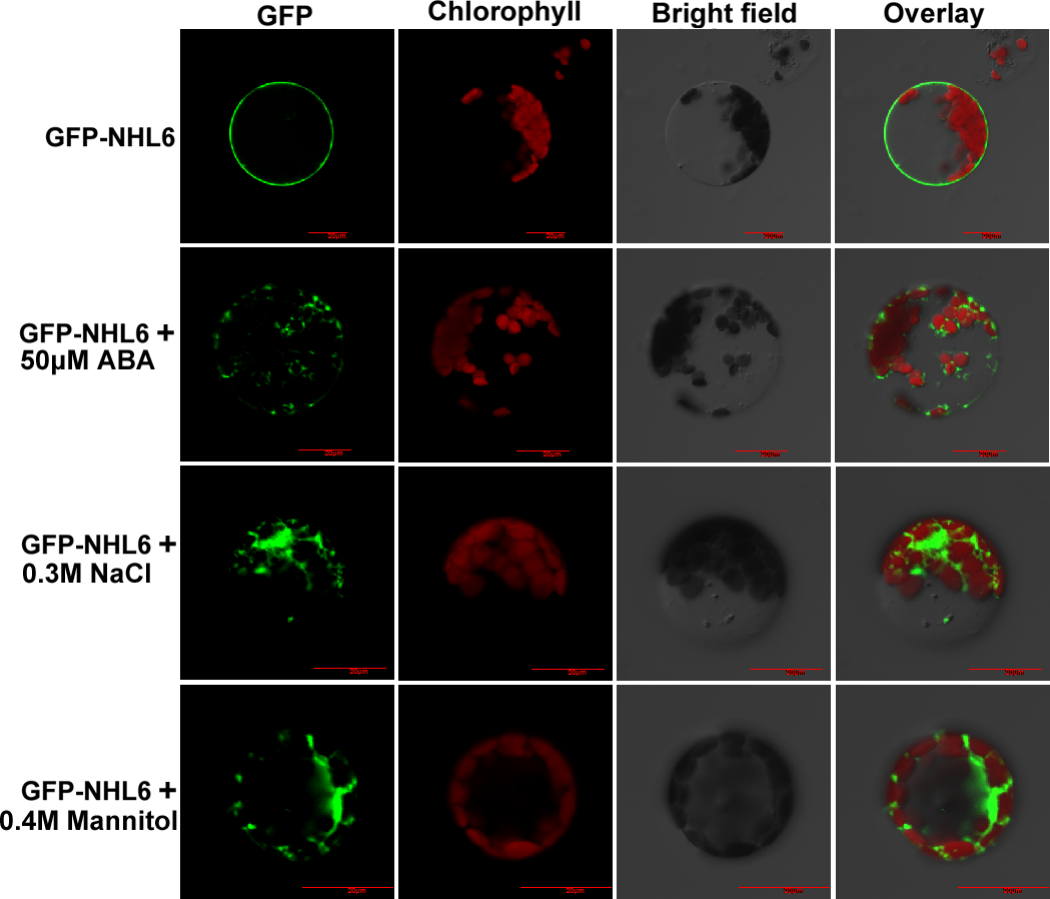
Osmotic-stress triggers the release of GFP-NHL6 from the plasma membrane. *Arabidopsis* protoplasts were transformed with GFP-NHL6 plasmid and incubated in dark at 23°C for 12 h. Protoplasts were then treated with 50 μM ABA, 0.3 M NaCl or 0.4 M mannitol for 1 h. Bar = 20μm.

NHL6 is predicted to be a plasma membrane localized protein with its N-terminal part in the cytoplasm (S2A Fig). This prediction was validated by the expression of YFP-NHL6 fusion protein in tobacco leaf epidermal cells (S2B Fig). Transient expression of GFP-NHL6 and GFP-AtTLP11, and co-expression of GFP-NHL6 and AtTLP11-mCherry (plasma membrane protein) in *Arabidopsis* protoplasts testified that GFP-NHL6 was localized to the plasma membrane (Fig 7A and 7B). Further analyses revealed that GFP-NHL6 is localized only to the plasma membrane under normal conditions. When treated with abiotic stress reagents such as ABA, NaCl and mannitol, GFP-NHL6 was released from the plasma membrane into the cytosol (Fig 8), but GFP-AtNSR3, a plasma membrane protein [33], was not (S4 Fig).

Previous studies have indicated that NHL family proteins may homo-dimerize probably to gain an active state [17,38]. Our BiFC experiments using YN-NHL and YC-NHL6 showed that indeed NHL6 also interacted with itself (S3 Fig). All these results demonstrate that NHL6 may play a function in abiotic stress response and ABA biosynthesis and/or signaling.

## Discussion

Although the functions of *NDR1/HIN1* and its homologous *NHL* gene family in disease associated biotic stress signaling have been extensively studied [14–18], little is known about their possible roles in ABA and abiotic stress-mediated response in plants. Here, we show that *NHL6*, a member of the *NHL* gene family, is involved in ABA signaling pathway during seed germination and early seedling development in *Arabidopsis*.

We observed that *NHL6* was predominantly expressed in seeds and senescent leaves, and its expression was strongly induced by ABA, mannitol and drought stress (Fig 1A and 1C). When we compared the germination of wild-type (Col-0), *nhl6* and *35S*::*NHL6* overexpression lines on MS medium supplemented with different concentrations of ABA, ABA-insensitive phenotypes were observed in *nhl6* (Fig 2), whereas *35S*::*NHL6* overexpression lines were hypersensitive to ABA, NaCl and mannitol (Figs 3C–3F and 4A–4D). The hypersensitivity of *35S*::*NHL6* overexpression lines to NaCl and mannitol can be largely due to the increased endogenous ABA accumulation. We observed that under high salt or hyperosmotic stress condition, more ABA was produced, which in turn probably inhibited the seed germination in transgenic plants. Application of ABA biosynthesis inhibitor norflurazon (NF) rescued the hypersensitivity to NaCl and mannitol in the transgenic plants reinforcing the idea that ABA was responsible for inhibition of seed germination (Fig 4E). Furthermore, we found higher ABA levels in both *35S*::*NHL6* overexpression lines 3 and 6 (Fig 4F), and the expression of several ABA biosynthesis genes was also highly induced by abiotic stress in the *35S*::*NHL6* overexpression plants (Fig 5). All these results suggest that the inhibited seed germination in transgenic plants was caused by the increased endogenous ABA accumulation.

ABA induced *NHL6* expression was dramatically restricted in *abi1-1C* (Fig 6A), and significantly blocked in the *areb1areb2abf3* triple mutant (Fig 6B). Our study with *ProNHL6*::LUC system showed that AREB1, AREB2 and ABF3 all induced the expression of LUC (Fig 6D), indicating that AREB1, AREB2 and ABF3 are required for the ABA-induced *NHL6* expression. Therefore, ABA signaling appears to be also involved in the ABA induced expression of *NHL6*, and the increased sensitivity to ABA in the *35S*::*NHL6* overexpression plants could be a result of both increased ABA production and altered ABA signaling.

The function of most proteins is closely related with their sub-cellular localizations. Consistent with the reported plasma membrane localization of NDR1, CaNDR1 and NHL3 [38,39,40], NHL6 is also localized to the plasma membrane, as confirmed by transient co-localization of GFP-NHL6 and mCherry-AtTLP11 in the *Arabidopsis* protoplasts (Fig 7). Previous studies have demonstrated that abiotic stresses such as salt and mannitol could translocate the GFP-tagged AtTLP3 Tubby domain (GFP-CT-AtTLP3Δ1–115) or GFP-AtTLP fusion protein from plasma membrane into the cytosol [33,41]. The observation that abiotic stress reagents could induce the translocation of GFP-NHL6 from plasma membrane to cytosol (Fig 8 and S4 Fig) indicates that NHL6 translocation responds to various reagents, implying that NHL6 could play a role in osmotic stress in *Arabidopsis*.

Late embryogenesis abundant (LEA) proteins, which accumulate at the late embryogenesis stage to counteract dehydration, were first discovered in cotton (*Gossypium hirsutum*) seeds [42]. LEA proteins are highly expressed when plants were subject to abiotic stress conditions such as salt, cold and drought [43,44]. The predicted structural homology of NDR1 to LEA14 underlies the potential role of NHL family proteins in abiotic stress responses [45]. As predicated using SMART [46,47], NHL6 harbors a LEA_2 domain at its C-terminal part. Coincidently, *NHL6* accumulates at high levels in the seeds, and its expression gradually decreases as the germination proceeds (Fig 1A and 1B), which might explain why no significant difference was observed when five-day-old seedlings of *nhl6* and *35S*::*NHL6* were transferred to high concentration of NaCl and mannitol (S5 Fig). Therefore, similar to the LEA proteins, NHL6 may also be required for the maintenance of seed dormancy under unfavorable conditions.

Taken together, our findings suggest that *NHL6* could function in one or several signal transduction pathway(s) by affecting ABA biosynthesis and signaling. Although the precise mode of the action of *NHL6* in plant response to ABA and abiotic stress is still intangible, the results of our study provide direct evidence that altered expression of *NHL6* can significantly modify ABA sensitivity and biosynthesis in transgenic plants. Further studies using genetic and biochemical strategies may help to open out how NHL6 collaboratively or counteractively works with itself or with other components to affect seed germination and early seedling development in *Arabidopsis*.

## Supporting Information

S1 Fig Microarray data of *NHL6* expression extracted from the *Arabidopsis* public database. (TIF)

S2 Fig Subcellular localization of NHL6 in *N*. *Benthamiana*. (TIF)

S3 Fig BiFC analysis of the self-interaction of NHL6 in *N*. *benthamiana*. (TIF)

S4 Fig Osmotic-stress does not trigger the release of GFP-AtNSR3 from the plasma membrane. (TIF)

S5 Fig Stress responses of *nhl6* and two *35S*::*NHL6* overexpressor lines to NaCl and mannitol. (TIF)

S1 Table Primers used in this study. (DOC)

## References

[1] Leung J, Giraudat J. Abscisic acid signal transduction. Annu Rev Plant Bio. 1998; 49: 199–222. 10.1146/annurev.arplant.49.1.199 15012233

[2] Finkelstein RR, Gampala SSL, Rock CD. Abscisic acid signaling in seeds and seedlings. Plant Cell. 2002: 14: S15–S45. 12045268 10.1105/tpc.010441 PMC151246

[3] Zhu JK. Salt and drought stress signal transduction in plants. Annu Rev Plant Bio. 2002; 53: 247–273. 12221975 10.1146/annurev.arplant.53.091401.143329 PMC3128348

[4] Nambara E, Marion-Poll A. Abscisic acid biosynthesis and catabolism. Annu Rev Plant Bio. 2005; 56: 165–185. 15862093 10.1146/annurev.arplant.56.032604.144046

[5] Leung J, Bouvier-Durand M, Morris PC, Guerrier D, Chefdor F, Giraudat J. *Arabidopsis* ABA response gene *ABI1*: features of a calcium-modulated protein phosphatase. Science. 1994; 264: 1448–1452. 7910981 10.1126/science.7910981

[6] Rodriguez PL, Benning G, Grill E. ABI2, a second protein phosphatase 2C involved in abscisic acid signal transduction in Arabidopsis. FEBS Lett. 1998; 421: 185–190. 9468303 10.1016/s0014-5793(97)01558-5

[7] Giraudat J, Hauge BM, Valon C, Smalle J, Parcy F, Goodman HM. Isolation of the Arabidopsis *ABI3* gene by positional cloning. Plant Cell. 1992; 4: 1251–1261. 1359917 10.1105/tpc.4.10.1251 PMC160212

[8] Finkelstein RR, Wang ML, Lynch TJ, Rao S, Goodman HM. The Arabidopsis abscisic acid response locus *ABI4* encodes an APETALA 2 domain protein. Plant Cell. 1998; 10: 1043–1054. 9634591 10.1105/tpc.10.6.1043 PMC144030

[9] Finkelstein RR, Lynch TJ. The Arabidopsis abscisic acid response gene *ABI5* encodes a basic leucine zipper transcription factor. Plant Cell. 2000; 12: 599–609. 10760247 10.1105/tpc.12.4.599 PMC139856

[10] Ma Y, Szostkiewicz I, Korte A, Moes D, Yang Y, Christmann A, et al Regulators of PP2C phosphatase activity function as abscisic acid sensors. Science. 2009; 324: 1064–1068. 10.1126/science.1172408 19407143

[11] Park SY, Fung P, Nishimura N, Jensen DR, Fujii H, Zhao Y, et al Abscisic acid inhibits type 2C protein phosphatases via the PYR/PYL family of START proteins. Science. 2009; 324: 1068–1071. 10.1126/science.1173041 19407142 PMC2827199

[12] Cutler SR, Rodriguez PL, Finkelstein RR, Abrams SR. Abscisic acid: emergence of a core signaling network. Annu Rev Plant Bio. 2010; 61: 651–679. 20192755 10.1146/annurev-arplant-042809-112122

[13] Yoshida T, Fujita Y, Sayama H, Kidokoro S, Maruyama K, Mizoi J, et al AREB1, AREB2, and ABF3 are master transcription factors that cooperatively regulate ABRE-dependent ABA signaling involved in drought stress tolerance and require ABA for full activation. Plant J. 2010; 61: 672–685. 10.1111/j.1365-313X.2009.04092.x 19947981

[14] Gopalan S, Bauer DW, Alfano JR, Loniello AO, He SY, Collmer A. Expression of the *Pseudomonas syringae* avirulence protein AvrB in plant cells alleviates its dependence on the hypersensitive response and pathogenicity (Hrp) secretion system in eliciting genotype-specific hypersensitive cell death. Plant Cell. 1996; 8: 1095–1105. 8768370 10.1105/tpc.8.7.1095 PMC161183

[15] Century KS, Shapiro AD, Repetti PP, Dahlbeck D, Holub E, Staskawicz BJ. NDR1, a pathogen-induced component required for *Arabidopsis* disease resistance. Science. 1997; 278: 1963–1965. 9395402 10.1126/science.278.5345.1963

[16] Dörmanna P, Gopalan S, He SY, Benning C. A gene family in *Arabidopsis thaliana* with sequence similarity to *NDR1* and *HIN1*. Plant Physiol Biochem. 2000; 38: 789–796.

[17] Zheng MS, Takahashi H, Miyazaki A, Hamamoto H, Shah J, Yamaguchi I, et al Up-regulation of *Arabidopsis thaliana NHL10* in the hypersensitive response to *Cucumber mosaic virus* infection and in senescing leaves is controlled by signalling pathways that differ in salicylate involvement. Planta. 2004; 218: 740–750. 14666423 10.1007/s00425-003-1169-2

[18] Varet A, Parker J, Tornero P, Nass N, Nurnberger T, Dangl JL, et al *NHL25* and *NHL3*, two *NDR1/HIN1-Like* genes in *Arabidopsis thaliana* with potential role(s) in plant defense. Mol Plant Microbe Interact. 2002; 15: 608–616. 12059109 10.1094/MPMI.2002.15.6.608

[19] Vilaine F, Kerchev P, Clement G, Batailler B, Cayla T, Bill L, et al Increased expression of a phloem membrane protein encoded by *NHL26* alters phloem export and sugar partitioning in *Arabidopsis*. Plant Cell. 2013 25: 1689–1708. 10.1105/tpc.113.111849 23715470 PMC3694700

[20] Pontier D, Gan SS, Amasino RM, Roby D, Lam E. Markers for hypersensitive response and senescence show distinct patterns of expression. Plant Mol Bio. 1999; 39: 1243–1255. 10380810 10.1023/a:1006133311402

[21] Zheng MS, Takahashi H, Miyazaki A, Yamaguchi K, Kusano T. Identification of the *cis*-acting elements in *Arabidopsis thaliana NHL10* promoter responsible for leaf senescence, the hypersensitive response against *Cucumber mosaic virus* infection, and spermine treatment. Plant Sci. 2005; 168: 415–422.

[22] Guo Y, Gan S. Leaf senescence: signals, execution, and regulation. Curr Top Dev Biol. 2005; 71: 83–112. 16344103 10.1016/S0070-2153(05)71003-6

[23] Schmid M, Davison TS, Henz SR, Pape UJ, Demar M, Vingron M, et al A gene expression map of *Arabidopsis thaliana* development. Nat Genet. 2005; 37: 501–506. 15806101 10.1038/ng1543

[24] Goda H, Sasaki E, Akiyama K, Maruyama-Nakashita A, Nakabayashi K, Ogawa M, et al The AtGenExpress hormone and chemical treatment data set: experimental design, data evaluation, model data analysis and data access. Plant J. 2008; 55: 526–542. 10.1111/j.0960-7412.2008.03510.x 18419781

[25] Antoni R, Gonzalez-Guzman M, Rodriguez L, Peirats-Llobet M, Pizzio GA, Fernandez MA, et al PYRABACTIN RESISTANCE1-LIKE8 plays an important Role for the Regulation of Abscisic Acid Signaling in Root. Plant Physiol. 2013; 161: 931–941. 10.1104/pp.112.208678 23370718 PMC3561030

[26] Murashige T, Skoog F. A revised medium for rapid growth and bio assay with tobacco tissue cultures. Physiol Plant. 1962; 15: 473–497.

[27] Liu H, Tang RJ, Zhang Y, Wang CT, Lv QD, Gao XS, et al AtNHX3 is a vacuolar K^+^/H^+^ antiporter required for low-potassium tolerance in Arabidopsis thaliana. Plant Cell Environ. 2010; 33: 1989–1999. 10.1111/j.1365-3040.2010.02200.x 20573049

[28] Bao Y, Wang CT, Jiang CM, Pan J, Zhang GB, Liu H, et al The tumor necrosis factor receptor-associated factor (TRAF)-like family protein SEVEN IN ABSENTIA 2 (SINA2) promotes drought tolerance in an ABA-dependent manner in *Arabidopsis*. New Phytol. 2014; 202: 174–187. 10.1111/nph.12644 24350984

[29] Clough SJ, Bent AF. Floral dip: a simplified method for Agrobacterium-mediated transformation of Arabidopsis thaliana. Plant J. 1998; 16: 735–743. 10069079 10.1046/j.1365-313x.1998.00343.x

[30] Jefferson RA, Kavanagh TA, Bevan MW. GUS fusion:betaglucurodinase as a sensitive and versatile gene fusion marker in higher plants. EMBO J. 1987; 6: 3901–3907. 3327686 10.1002/j.1460-2075.1987.tb02730.x PMC553867

[31] Hellens RP, Allan AC, Friel EN, Bolitho K, Grafton K, Templeton MD, et al Transient expression vectors for functional genomics, quantification of promoter activity and RNA silencing in plants. Plant Methods. 2005; 1: 13 16359558 10.1186/1746-4811-1-13 PMC1334188

[32] Yoo SD, Cho YH, Sheen J. Arabidopsis mesophyll protoplasts: a versatile cell system for transient gene expression analysis. Nat Protoc. 2007; 2: 1565–1572. 17585298 10.1038/nprot.2007.199

[33] Bao Y, Song WM, Jin YL, Jiang CM, Yang Y, Li B, et al Characterization of *Arabidopsis* Tubby-like proteins and redundant function of AtTLP3 and AtTLP9 in plant response to ABA and osmotic stress. Plant Mol Biol. 2014; 86: 471–481. 10.1007/s11103-014-0241-6 25168737

[34] Zhu JQ, Zhang JT, Tang RJ, Lv QD, Wang QQ, Yang L, et al Molecular characterization of *ThIPK2*, an inositol polyphosphate kinase gene homolog from *Thellungiella halophila*, and its heterologous expression to improve abiotic stress tolerance in *Brassica napus*. Physiol Plant. 2009; 136: 407–425. 10.1111/j.1399-3054.2009.01235.x 19470090

[35] Yang L, Tang RJ, Zhu JQ, Liu H, Mueller-Roeber B, Xia HJ, et al Enhancement of stress tolerance in transgenic tobacco plants constitutively expressing AtIpk2 beta, an inositol polyphosphate 6-/3-kinase from Arabidopsis thaliana. Plant Mol Biol. 2008; 66: 329–343. 10.1007/s11103-007-9267-3 18165921 PMC2238787

[36] Seo M, Koshiba T. Complex regulation of ABA biosynthesis in plants. Trends Plant Sci. 2002; 7: 41–48. 11804826 10.1016/s1360-1385(01)02187-2

[37] Lescot M, Dehais P, Thijs G, Marchal K, Moreau Y, Van de Peer Y, et al PlantCARE, a database of plant cis-acting regulatory elements and a portal to tools for in silico analysis of promoter sequences. Nucleic Acids Res. 2002; 30: 325–327. 11752327 10.1093/nar/30.1.325 PMC99092

[38] Varet A, Hause B, Hause G, Scheel D, Lee J. The Arabidopsis *NHL3* gene encodes a plasma membrane protein and its overexpression correlates with increased resistance to *Pseudomonas syringae* pv. *tomato* DC3000. Plant Physiol. 2003; 132: 2023–2033. 12913158 10.1104/pp.103.020438 PMC181287

[39] Coppinger P, Repetti PP, Day B, Dahlbeck D, Mehlert A, Staskawicz BJ. Overexpression of the plasma membrane-localized NDR1 protein results in enhanced bacterial disease resistance in Arabidopsis thaliana. Plant J. 2004; 40: 225–237. 15447649 10.1111/j.1365-313X.2004.02203.x

[40] Cacas JL, Petitot AS, Bernier L, Estevan J, Conejero G, Mongrand S, et al Identification and characterization of the Non-race specific Disease Resistance 1 (NDR1) orthologous protein in coffee. BMC Plant Biol. 2011; 11: 144 10.1186/1471-2229-11-144 22023696 PMC3212813

[41] Reitz MU, Bissue JK, Zocher K, Attard A, Huckelhoven R, Becker K, et al The Subcellular Localization of Tubby-Like Proteins and Participation in Stress Signaling and Root Colonization by the Mutualist Piriformospora indica. Plant Physiol. 2012; 160: 349–364. 10.1104/pp.112.201319 22751378 PMC3498949

[42] Galau GA, Hughes DW, Dure L. Abscisic-acid induction of cloned cotton late embryogenesis-abundant (Lea) mRNAs. Plant Mol Biol. 1986; 7: 155–170. 10.1007/BF00021327 24302301

[43] Bray EA. Plant responses to water deficit. Trends Plant Sci. 1997; 2:48–54.

[44] Thomashow MF. Plant cold acclimation: Freezing tolerance genes and regulatory mechanisms. Annu Rev Plant Physiol Plant Mol Biol. 1999; 50: 571–599. 15012220 10.1146/annurev.arplant.50.1.571

[45] Knepper C, Savory EA, Day B. Arabidopsis NDR1 is an integrin-like protein with a role in fluid loss and plasma membrane-cell wall adhesion. Plant Physiol. 2011; 156: 286–300. 10.1104/pp.110.169656 21398259 PMC3091050

[46] Schultz J, Milpetz F, Bork P, Ponting CP. SMART, a simple modular architecture research tool: Identification of signaling domains. Proc Natl Acad Sci USA. 1998; 95: 5857–5864. 9600884 10.1073/pnas.95.11.5857 PMC34487

[47] Letunic I, Doerks T, Bork P. SMART 7: recent updates to the protein domain annotation resource. Nucleic Acids Res. 2002; 40: D302–D305. 10.1093/nar/gkr931 PMC3245027 22053084

