# Supplementary figures and images for: Overexpression of the NDR1/HIN1-Like Gene NHL6 Modifies Seed Germination in Response to Abscisic Acid and Abiotic Stresses in Arabidopsis
Source: PLoS One. 2016 Feb 5;11(2):e0148572. doi: 10.1371/journal.pone.0148572 (PMC4744021; doi:10.1371/journal.pone.0148572)

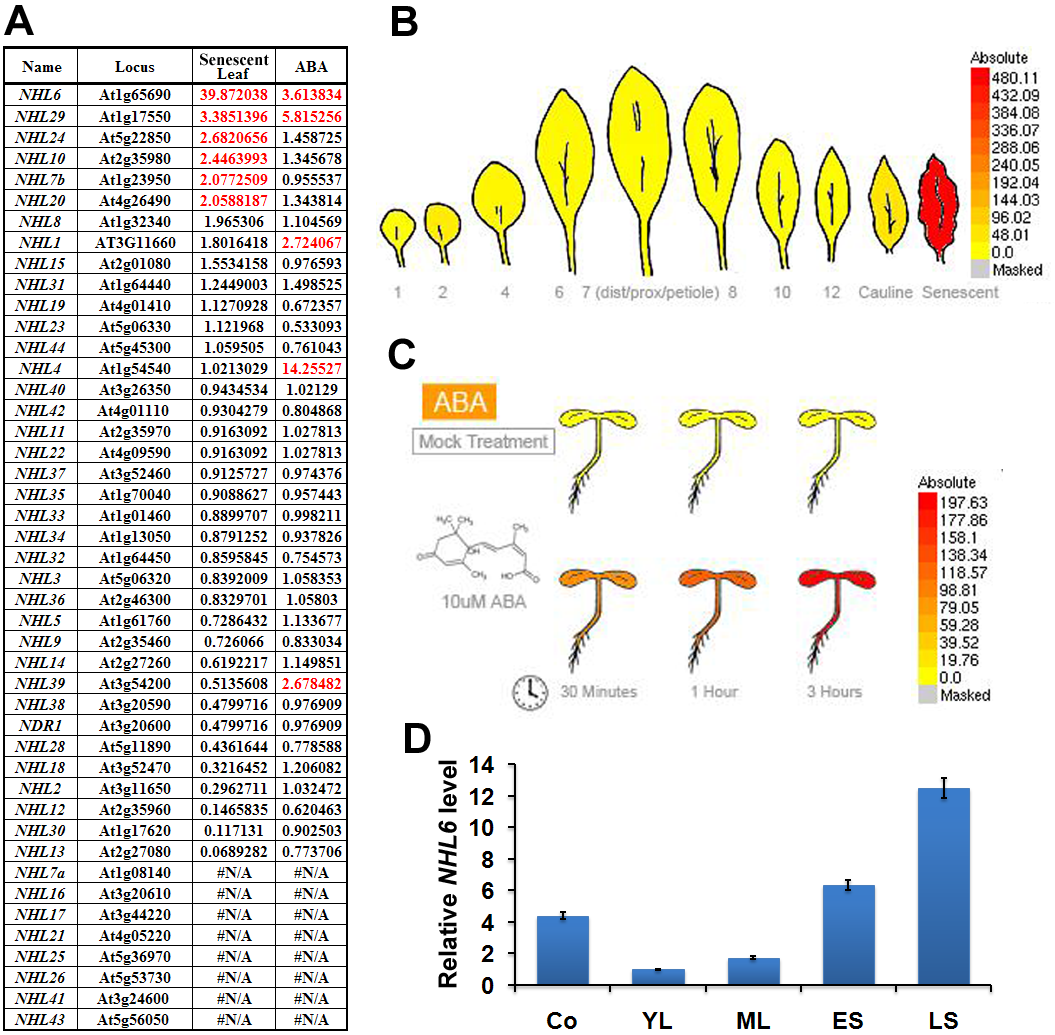

Supplement: S1 Fig — (TIF) [file pone.0148572.s001.tif]

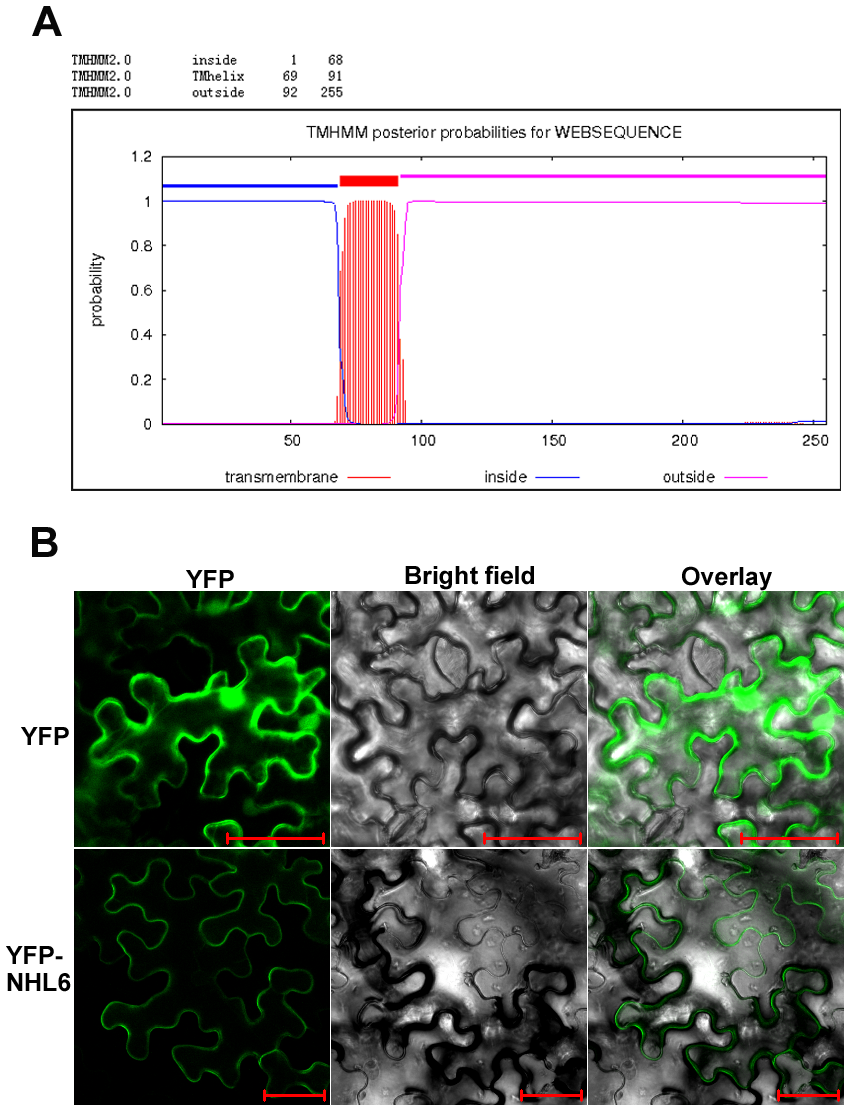

Supplement: S2 Fig — (TIF) [file pone.0148572.s002.tif]

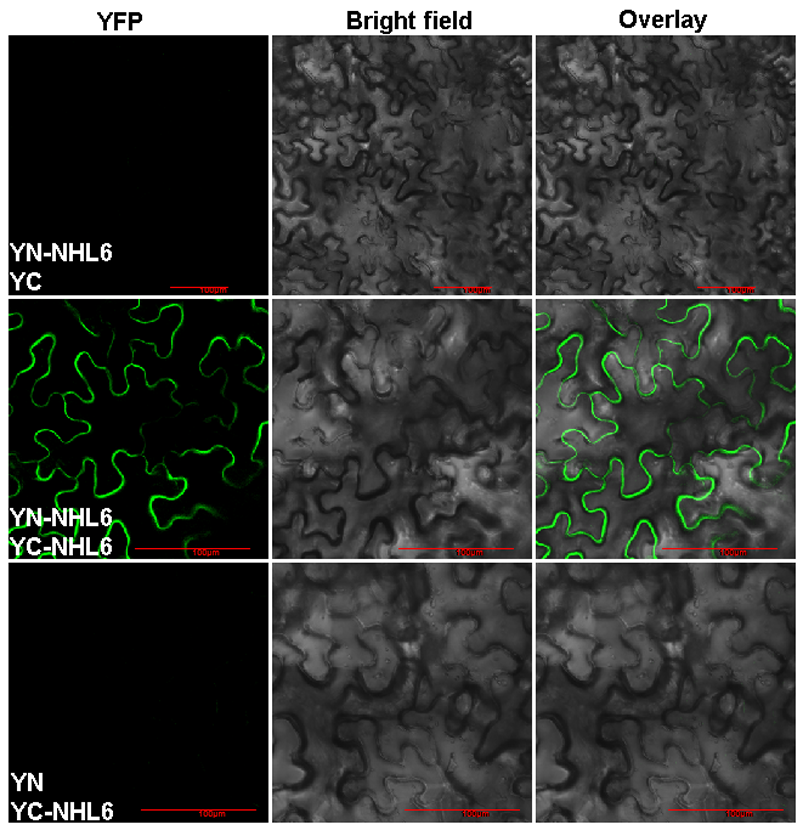

Supplement: S3 Fig — (TIF) [file pone.0148572.s003.tif]

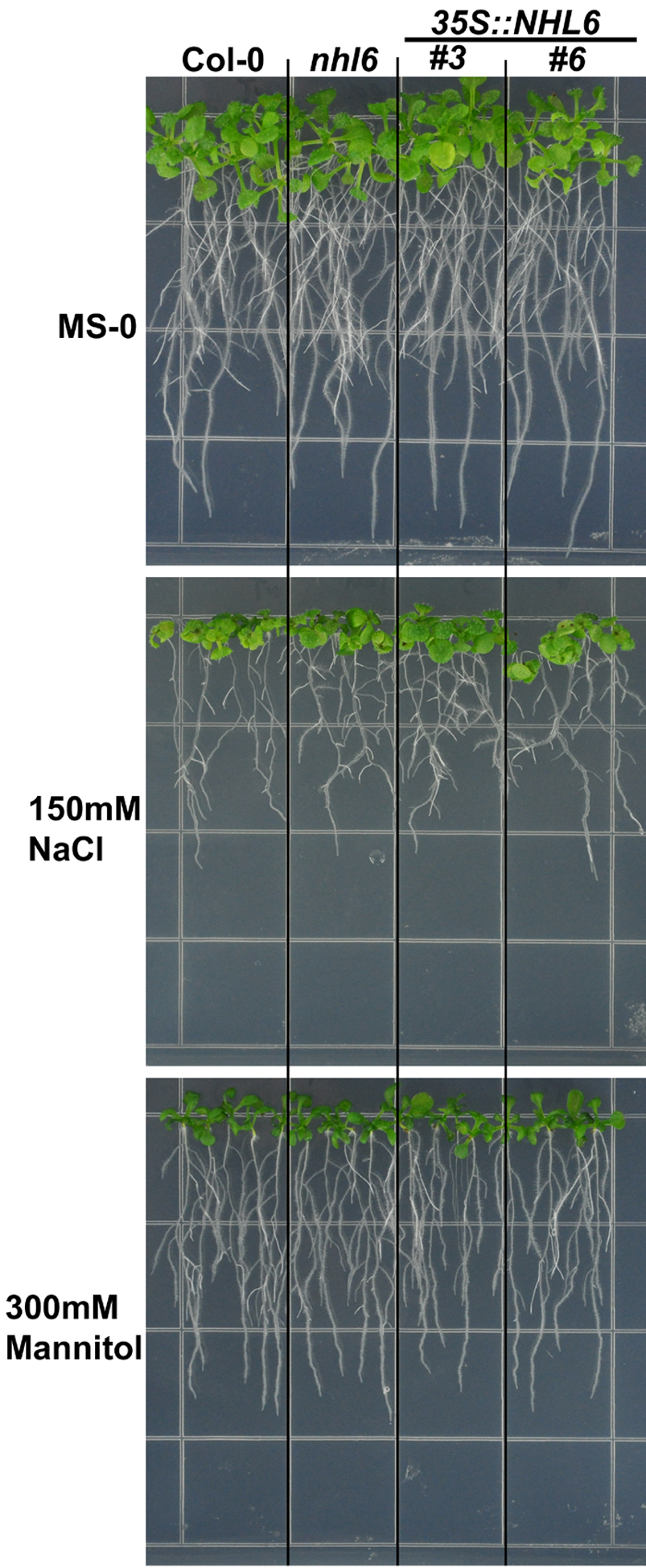

Supplement: S4 Fig — (TIF) [file pone.0148572.s004.tif]

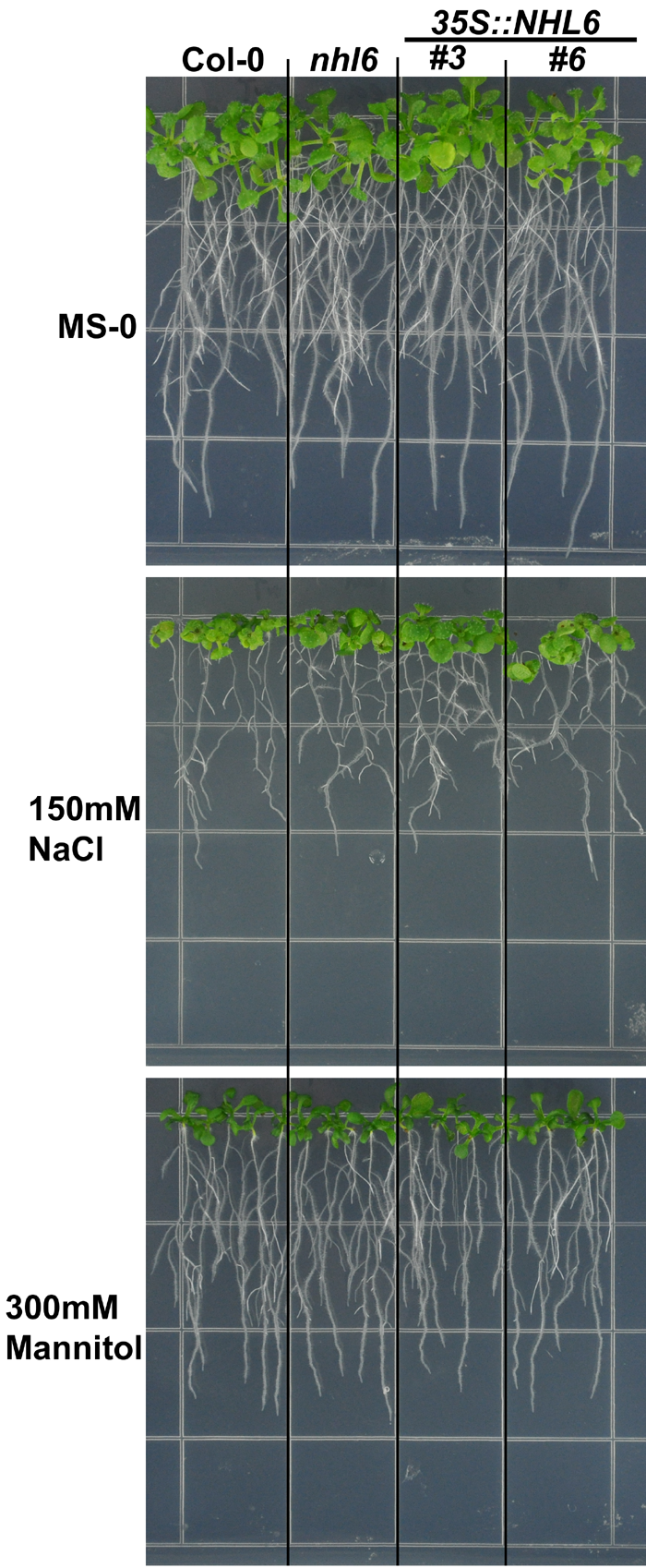

Supplement: S5 Fig — (TIF) [file pone.0148572.s005.tif]
