# Supplementary material for: Overexpression of the NDR1/HIN1-Like Gene NHL6 Modifies Seed Germination in Response to Abscisic Acid and Abiotic Stresses in Arabidopsis
Source: PLoS One. 2016 Feb 5;11(2):e0148572. doi: 10.1371/journal.pone.0148572 (PMC4744021; doi:10.1371/journal.pone.0148572)
Supplement: S1 Table — (DOC) [file pone.0148572.s006.doc]

| **S1 Table. Primers used in this study** | |
| --- | --- |
| For gene cloning |  |
| Primer name | Primer sequence |
| PrNHL6F | 5'TCTCATTCAAAGCCTAAAAACAA 3' |
| PrNHL6R | 5'TTTTGGAAAATCTTGATGACACC 3' |
| gNHL6R | 5'GCGTTTCTTTTCTACATGTCTCTC 3' |
| NHL6F | 5'ATGTCTCAACACCAAAAAATCTATCC 3' |
| NHL6R | 5'CTATAACCTAAGACGAAATTTGCAA 3' |
| NHL6NR+*Sal*I | 5'ccggtcgacCTAAAGAAAGCAGAACGTGTAGCA 3' |
| ABI5F+ *Bam*HI | 5'gccgggatccATGGTAACTAGAGAAACGAAGTTGA 3' |
| ABI5R+ *Sal*I | 5'tcagtcgacTTAGAGTGGACAACTCGGGTT 3' |
| ABF3F+ *Bam*HI | 5'gccgggatccATGGGGTCTAGATTAAACTTCAAG 3' |
| ABF3R+ *Sal*I | 5'tcagtcgacCTACCAGGGACCCGTCAAT 3' |
| AREB1F+ *Bam*HI | 5'gccgggatccATGGATGGTAGTATGAATTTGGG 3' |
| AREB1R+ *Sal*I | 5'tcagtcgacTCACCAAGGTCCCGACTCT 3' |
| AREB2F+ *Bam*HI | 5'gccgggatccATGGGAACTCACATCAATTTCAA 3' |
| AREB2R+ *Sal*I | 5'tcagtcgacTCACCATGGTCCGGTTAATG 3' |
| For qRT-PCR and RT-PCR | |
| Primer name | Primer sequence |
| RT-NHL6F | 5'AACCGTACAATTGTCCACACTCAGG 3' |
| RT-NHL6R | 5'TTCAGCTCCTTGTCGGTGGTGTT 3' |
| ACTIN2F | 5'GGAAGGATCTGTACGGTAAC 3' |
| ACTIN2R | 5'GGACCTGCCTCATCATACT 3' |
| NCED3F | 5' TGGCTTCTTTCACGGCAACG 3' |
| NCED3R | 5' CGGACGGGCTGTTCATTCAC 3' |
| AAO2F | 5' TGTCATGAAAAACGCGTACTCTCT 3' |
| AAO2R | 5' CGCAGTGCACCGAAGCT 3' |
| AAO3F | 5' GAAGGTCTTGGAAACACGAAGAA 3' |
| AAO3R | 5' GAAATACACATCCCTGGTGTACAAAAC 3' |
| ABA3F | 5' TCCTGAAGATTACAGTTGCTTATTCAC 3' |
| ABA3R | 5' TGGGTCCACGGAAAAGTCTCT 3' |
| *Letters in lower case are protective cases or restriction sites | |
